# Personality Attributions in the Context of Stalking of Ex-Intimates or Others: A Victim’s Perspective

**DOI:** 10.1177/0306624X241240703

**Published:** 2024-03-21

**Authors:** Lily Truss, Melissa S. de Roos

**Affiliations:** 1 University of Roehampton, London, UK; 2 Erasmus University Rotterdam, The Netherlands

**Keywords:** stalking, victimization, personality, intimate partner violence, control

## Abstract

Stalking perpetrators may suffer from maladaptive personality traits, particularly if they stalk in the context of an (ex-)intimate relationship. To date, no study has examined how different personality attributions may relate to stalker motivation, or the behaviors they engage in, and how this differs across victim-perpetrator relationships. Further, the perspective of the victim is often not taken into consideration, even though most stalking victims know their stalker intimately and a majority are stalked by a former or current partner. The present study employed a correlational design to assess the relationship between stalking behaviors, motivation to stalk, and personality attributions, as perceived by the victim across an ex-intimate or other victim-perpetrator relationship. The study sample consisted of 100 victims of stalking (63% ex-intimate; 85% female) who were recruited through a National Stalking Helpline. Results align with and extend the results of previous researchers, most notably the high proportion of reported Cluster B-aligned personality attributions among stalkers, as well as the proportion of more under-researched personality attributions, and their associated risks. Victims of an ex-intimate partner were more likely to report their stalker was motivated by intimacy, and personality attributions aligned with both borderline and paranoid PD were more often reported than in other relationship contexts. Results and clinical implications are discussed.

Intimate partner violence (IPV) remains a persistent problem. The Crime Survey for England and Wales estimated that 5.5% of adults over the age of sixteen had experienced IPV in the previous year; a number that translates to 2.3 million people (Office for National Statistics [ONS], 2020). Increasingly, the overlap between stalking and intimate partner violence has been highlighted (Douglas & Dutton, 2001; McEwan et al., 2017). Indeed, the Office for National Statistics reports stalking by a current or ex-partner as part of their IPV statistics. When looking at stalking statistics, it is estimated that nearly 5% of women, and 2.4% of men in the UK experienced stalking in the past year (ONS, 2019). Between 40% and 50% of these cases involved stalking by an (ex-) intimate partner. When including cases of casual dating, this percentage rises to almost 70%. On the other hand, in the United States a slight decline in prevalence was reported in the between 2016 and 2019 (1.5%–1.3%; Morgan & Truman, 2022). Here, people were most often stalked by an acquaintance, with almost 25% stalked by an (ex-) intimate partner. However, when assessing risk factors for physical harm resulting from violent stalking, a prior intimate relationship consistently emerges as one of the strongest risk factors (e.g., Churcher & Nesca, 2013). Indeed, at its extreme, stalking shows a consistent association with femicide (Brady & Hayes, 2018; Matias et al., 2020; Rai et al., 2020; Spencer & Stith, 2020).

Although there is some disagreement over what constitutes stalking, it can best be defined as two or more incidents of repeated and unwanted communication, contact or other conduct that cause significant emotional distress and/or fear for the safety of the victim or others (Mullen et al., 2001;Van Der Aa, 2018). This conduct is motivated by obsession or fixation with the victim, and the perpetrator deliberately or recklessly causes the victim to experience fear or concern for their safety or the safety of others (Monckton-Smith et al., 2017). Example behaviors include but are not limited to, loitering outside the victim’s home, phone calls, emails, notes, or in extreme cases, assault, or even murder (Kamphuis & Emmelkamp, 2000). Extreme harm or murder is most likely to occur if the victim is female, and particularly in the context of an (ex-) romantic relationship (McFarlane et al., 2002).

Stalking often results in negative psychological and social consequences sustained by the victims, mainly from prolonged, unwanted, and unpredictable intrusions that produce a sense of powerlessness (Kamphuis & Emmelkamp, 2000). Consequences may be psychological, social, or economic (e.g., Sheridan & Grant, 2007). A recent study explored victims’ experiences of stalking with reports of various adverse psychological effects such as anxiety and depression, post-traumatic stress disorder (PTSD), suicide ideation, eating disorders and substance abuse (Taylor-Dunn et al., 2021). Those who are stalked in the context of an (ex-) romantic relationship experience greater levels of fear (e.g., Logan & Walker, 2017) which may reflect the greater likelihood of stalking in this context escalating to violence. Further, it appears that the duration of stalking itself is associated with PTSD symptoms in some victims, with victims who were stalked for longer reporting more symptoms (Kamphuis et al., 2003). These findings highlight the importance of early intervention, for example through police involvement or victim support to prevent or put a stop to persistent stalking.

Unfortunately, victims of stalking face several obstacles when choosing to involve the police. Victims often feel they are not being taken seriously (Korkodeilou, 2014) or they view the police as unhelpful (Galeazzi et al., 2009). Victims have reported police telling them to change their own behavior, such as take alternate routes, or to warn their perpetrators, both of which can be ineffective and may actually increase stalking behaviors among obsessed and fixated individuals (Meloy, 1999; Ostermeyer et al., 2016). It seems that although recent developments place a greater emphasis on protecting the victim by law, it may take more time to recognize the severity of stalking and the associated risks in practice (Taylor-Dunn et al., 2021). Hence, more research is needed to understand the motivations, maintaining factors and risks associated with stalking, particularly in the context of a past or current romantic relationship. This information can then be used to provide up to date training to police and victim support, to aid in recognizing the situation and the risks as well as to support the victim.

## Stalking Motivations

Research has attempted to create various typologies of stalkers (e.g., Mohandie et al., 2006; Mullen et al., 1999) based on factors such as relationship between victim and perpetrator or motivations of the stalker. However, more recently, research suggests there is substantial overlap between subtypes of stalkers, and we cannot speak of distinct categories (e.g., Youngs et al., 2013), let alone base predictions on such categories. Rather, a broader focus may be on what motivates people to stalk, and what sort of behaviors are related to this.

Motivations to stalk are diverse. A recent study by Chan (2021) found the three most common self-reported motivations of stalking perpetrators were finding the victim attractive, getting the victim back into a relationship, and controlling the victim. Increasingly, attention is paid to victims’ perceptions of stalker motivation. In a large survey (*n* = 2768) on the impact of cyberstalking, victims reported perceived motivations of reaction to a real or imagined rejection, or insult or injury by the victim (Dreßing et al., 2014). Jealousy, a desire to start a romantic relationship, and revenge were also cited as motivations. Similarly, Fissel (2022) asked 576 cyberstalking victims about their perceptions of the perpetrator’s motivation. Participants most commonly thought their stalker was motivated by affection, rejection, or obsession. Further, Fissel found that if the perceived motive was retaliation, revenge, or rejection, participants were most likely to be fearful, whereas the opposite was true for affection-related motivations. These patterns held after controlling for seriousness of offense and demographics. These findings highlight the importance of exploring how victims perceive the motivations of their stalkers, as this perception affects their wellbeing. Indeed, perceived motivations have been linked to harm suffered by the victim. Randa et al. (2022) used the supplemental stalking surveys to the National Crime Victimization Survey to assess perceived motivations. Most participants (70.7%) selected power and control as at least one of their stalker’s motivations, followed by relational, denoting motivation following from romantic failure (69%). The final motivation was arrogance, reported by 55% of participants. The motivation most strongly related to harm reported by the victim was power and control, followed by the motivation that the perpetrator stalked simply because they felt that they could get away with it. A relational motivation was not significantly related to harm, which naturally does not indicate this form of stalking is not harmful. It appears then that perceived motivations affect both fearfulness of the victim as well as broader harm experienced.

## Personality Disorders

Personality disorders (PD’s) are characterized by an enduring pattern of inner experience and behavior that deviates markedly from the expectations of the individual’s culture (DSM-5: American Psychiatric Association, 2013). The current edition of the DSM identifies 10 PD’s organized in three clusters. Personality disorders in Cluster A are characterized as odd and eccentric. People with such disorders tend to be introverted, detached from others, and paranoid (e.g., Kosson et al., 2008). Cluster A PD’s are schizoid, schizotypal, and paranoid disorders. Cluster B encompasses the dramatic, emotional, and erratic disorders, characterized by unstable interpersonal relationships and increased reactivity to stress (e.g., Wingenfeld et al., 2010). Personality disorders in this cluster are antisocial, borderline, histrionic, and narcissistic. Finally, Cluster C represents anxious, fearful PD’s, namely avoidant, dependent, and obsessive-compulsive personality disorders.

Personality disorders have long been a focus for research into stalking perpetration. In the study by Mullen et al. (1999) the primary diagnosis among perpetrators was a PD. Cluster B PD’s were the most prevalent of these diagnoses, and these perpetrators were at an increased risk of escalation to violent behavior. Indeed, the majority of research into links between stalking and PD’s has focused exclusively on cluster B PD’s (Meloy et al., 2000; Rosenfeld, 2003; Sansone & Sansone, 2010). However, stalking is not exclusively based on predatory or violent motives typically associated with Cluster B PD’s (Spitzberg & Cadiz, 2002). As such, more research is necessary to explore other PD characteristics among stalking perpetrators. For example, it is plausible that people with Dependent PD who worry excessively about being alone (De Francisco Carvalho et al., 2019) may resort to stalking when someone leaves them in order to retain intimacy. Thus, while previous research has provided an understanding into the rates of cluster B PD’s among stalkers, PDs within other clusters tend to be neglected. An exception is a study by McEwan et al. (2017), who found that personality disorders were more common among ex-intimate stalkers than acquaintance or stranger stalkers, with Cluster B PDs most commonly reported across both groups.

However, these studies rely on data from clinical and forensic samples. This may explain why research has found evidence of cluster B PDs in stalking perpetrators, but not other PDs; individuals who have been arrested and detained are more likely to be emotionally dysregulated and violent individuals (Mullen et al., 1999). It is plausible that more conscientious individuals such as those with Obsessive Compulsive PD features may not be arrested or convicted.

Indeed, studies with community samples still highlight a high prevalence of PD among stalking perpetrators (Nijdam-Jones et al., 2018; Spitzberg & Veksler, 2007). Nijdam-Jones and colleagues broadened the scope to include paranoid PD and other PDs. No significant differences were found between stalking offenders with and without cluster B PD’s (such as the relationship to the victim and their motivation for stalking). This finding highlights the possibility that those without a diagnosis of PD may be missing a diagnosis or may still have high rates of PD features that may be associated with their stalking behaviors and motivations for stalking. As such, an understanding of what leads individuals to perpetrate stalking requires more than evaluating whether a clinical diagnosis is warranted.

Similarly, Spitzberg and Veksler (2007) explored stalking behaviors experienced in a community sample. They asked self-identified victims of stalking to identify the stalkers’ personality traits, as opposed to asking the perpetrators themselves. This study emphasized the experience of the victim, which as highlighted previously, is often misunderstood or not taken seriously. Further, victims will be less likely to display a social desirability bias than perpetrators (Edwards, 1957) and they provide a unique perspective on the behavior of the perpetrator.

Despite these strengths, Spitzberg and Veksler’s (2007) research is not without its limitations. The participants were not provided with a definition of stalking and participants’ definitions of stalking have been demonstrated to vary from legal definitions. For example, 30.7% of those who reported being stalked had actually experienced harassment and 46% reported they had been harassed, but in legal terms, their experience constituted stalking (Spitzberg & Veksler, 2007). Thus, the sample consisted of potentially unacknowledged victims of stalking, as well as participants who were victims of a different type of crime. The present study takes this into account by using the legal definition of stalking in the UK, which was provided to the participants to establish whether they were a victim of stalking, at the time they emailed into the helpline for advice.

## The Present Study

Personality disorders appear to be prevalent among stalking perpetrators, particularly ex-intimate stalkers. The emphasis has been on Cluster B PDs, typically assessed in a clinical population. Less attention has been given to nonclinical samples, with a broader focus on personality attributions beyond those linked to Cluster B PDs. Further, there is a need for greater insight into the victim’s perceptions of the stalking situation, to inform early intervention strategies.

The present study aims to explore the association between personality attributions and motivation to stalk as perceived by the victim. We are particularly interested in how such a link may manifest in people who stalk a current or ex-partner compared with another type of victim-perpetrator relationship. Although this study is exploratory, we expect to find some specific relations. We hypothesize that personality attributes associated with Cluster A and C PDs will be linked to a different motivation to stalk than personality attributes more closely aligned with Cluster B PDs. Further, we expect to see a distinction in the types of stalking behaviors engaged in, based on the stalker’s motivation and victim-perpetrator relationship. Previous research by Youngs et al. (2013) has indicated that alternative psychological frameworks are needed for understanding the detailed behaviors of stalkers. Thus, we are interested in examining whether there is also a relationship between motivation to stalk, behaviors, and personality disorders, considering the overlap between stalking subtypes found in their study. Our hypotheses were not preregistered.

The present study was conducted with a non-clinical sample, thus capturing a greater variety of stalking behaviors than forensic or clinical samples. In doing so, this study allows an exploration into stalkers that defy the stereotypes of stalkers as a “psychopathic and violent” and focus on a broader experience of stalking that may include “less serious” behaviors (Spitzberg & Cadiz, 2002), particularly those experienced in the context of a current or past intimate relationship. These behaviors are specifically important to explore, as they provide a focal point for early interventions aimed at preventing escalation. Moreover, the present study will focus on the victims’ perspectives of stalking, to help us gain an insight into the motivation to stalk, severity of stalking and the associated risks from a unique perspective. The Stalking and Harassment Risk Identification Checklist (S-DASH; stalking version of DASH, Richards, 2009) lists victim fear as a risk factor for future violent stalking behaviors, highlighting the importance of gaining an insight into the victims perspective of motivations for stalking.

## Method

### Participants

Participants were recruited through the National Stalking Helpline, part of a charity organization in the United Kingdom that aims to educate and campaign to reduce the risk and prevalence of stalking and to support victims. They provide support and advice, including information on how to stay safe and report stalking. Victims of stalking may be provided contact details for the helpline by police, and various websites (including the government’s website) point to the helpline if people think they are being stalked.

The first author conducted an internship at the helpline and collected the data. Via email, we contacted approximately 500 self-identified victims of stalking who had contacted the helpline in the past year and who had also indicated they were willing to participate in research. All of these victims had indicated they were stalked by someone they knew, rather than by a stranger. Initially, 154 people accessed the survey. We removed people who filled out <75% of the survey (*n* = 53) and one participant who was underage. This resulted in a final sample of 100 participants (response rate ≈ 20%; 85% female). Although the majority of our victim sample identified as female, it is important to note that women can be perpetrators, and men can be victims. Further, seeing as we did not ask participants the perpetrator gender, we cannot, and indeed should not, assume that most perpetrators in this sample were male. The age of participants ranged from 18 to 70 years old (*M* = 41.18, *SD* = 11.88). Due to the sensitive nature of this data and the recruitment of participants through a helpline, we are not able to make the data openly available. Ethical approval was obtained from the university ethics board.

### Procedure

Participants were contacted via email, and provided with a link to the Qualtrics survey. Participants were asked to sign a consent form, and they were informed that their participation was anonymous. The three questionnaires were filled out in Qualtrics, and basic demographic info was collected (age, gender, relationship to the perpetrator, duration of stalking perpetration). Participants had the option of skipping any questions they did not want to answer. At the end of the survey, participants were provided with a debrief form and resources for them to contact if they felt the need to speak with someone after participation.

### Materials

#### Stalking behaviors

Stalking behaviors were assessed through a checklist that comprised 20 stalking behaviors the victims may have experienced at the hands of the perpetrator. These stalking behaviors were divided into five groups: a) communication, such as text messages or phone calls; b) violence, such as physical assault or property damage; c) proximity, such as following or showing up at the victim’s home; d) monitoring, such as the use of tracking devices or digital surveillance; and e) other, such as making false allegations or unwanted gifts. Participants responded to each item with yes or no to indicate whether they had experienced the behavior. Items were then summed to provide a total score for each of the five groups, with higher scores indicating more behaviors in that category.

#### Personality attributions

To assess victims’ perceptions of their stalker’s personality attributions, a questionnaire based on the DSM-5 criteria for each PD was developed. Each PD has six to nine symptoms listed in the DSM. We rewrote these symptoms into questionnaire items, ensuring that the language was accessible. For example, the borderline PD symptom: “Frantic efforts to avoid real or imagined abandonment” was rewritten as “Does this person make extreme efforts to avoid real or imagined abandonment?” This method is consistent with Spitzberg and Veksler (2007). The final questionnaire consisted of 74 questions, which were scored on a 5-point Likert scale (1 = *Never*, 5 = *Always*). Sample items and descriptive statistics are displayed in Table 2. Mean scores were calculated for each subscale. The reliability of each subscale exceeded *α* = .75, except for schizotypal (*α* = .70) and schizoid (*α* = .72) personality attributions.

#### Stalking motivations

To assess the motivation for stalking, we constructed a thirteen-item questionnaire loosely based on research outlined by Mullen et al. (1999). Each item was scored on a 5-point Likert scale (1 = *Definitely yes*, 5 = *Definitely no*). We conducted an Exploratory Factor Analysis on the 13 items to assess stalking motivations, using Principal Component Analysis with a Oblimin rotation and Kaiser Normalization. Results are displayed in Table 1. Five factors with were extracted based on the scree plot and eigenvalues >1. The second extracted factor appeared to relate to an aggrieved perpetrator, which we felt did not align with motivations as clearly as the two chosen factors. The third one related more to the how the perpetrator perceived the victim’s lack of interest. We used theoretical considerations in conjunction with factor loadings to select the final items, choosing factors 1 and 5, which were characterized by Desire for Intimacy (Factor 1) and Desire to Frighten (Factor 2).

**Table 1.** Rotated Component Matrix of Exploratory Factor Analysis of Stalking Motivations Items.

Item	Factor				
	1	2	3	4	5
1. Does/did this person desire a relationship with ‘true love’?	**.89**				
2. Is this person oblivious to victim’s responses?	.22	.35	−.58	−.16	
3. Does/did this person think that you are in love with them?	**.85**				
4. Does/did this person view you with special desirability?	**.69**			.23	−.18
5. Does/did this person acknowledge victim’s disinterest?		.20	.81		.15
6. Does/did this person stalk with the hope of intimacy?	**.62**			.43	−.18
7. Was this person initially only interested in a brief relationship?				.91	
8. Does/did this person feel persecuted or mistreated?		.90			
9. Is/was this person intending to frighten or distress?			.22		**.78**
10. Does/did this person present as the victim?		.89			
11. Does this person enjoy the sense of control that comes from stalking?	.18				**.90**
12. Does/did this person stalk to observe the victim?			−.55		**.43**
13. Does/did this person ever attack without warning?	−.23	.22	−.29		**.38**

*Note*. Bolded factors were selected for Confirmatory Factor Analysis. Loadings of <.15 are not displayed.

We then performed a Confirmatory Factor Analysis specifying this two-factor model. The result was an acceptable fit to data for most fit indices (*X*^2^ = 32.654, *p* = .10, RMSEA = .09, CFI = .928, TLI = .894, SRMR = .07). Factor loadings are displayed in Table 2. These eight items were used to assess stalking motivation.

**Table 2.** Confirmatory Factor Analysis Factor Loadings.

Item	Factor	
	1	2
Does/did this person desire a relationship with “true love”?	.84	
Does/did this person think that you are in love with them?	.68	
Does/did this person view you with special desirability?	.71	
Does/did this person stalk with the hope of intimacy?	.60	
Is/was this person intending to frighten or distress?		.71
Does this person enjoy the sense of control that comes from stalking?		.72
Does/did this person stalk to observe the victim?		.43
Does/did this person ever attack without warning?		.45

### Statistical Analyses

All statistical analyses were conducted in SPSS version 28.0.1.0. Prior to analyses, data were screened and participants were removed as detailed in the participants section. There were no significant deviations from assumptions. Due to the relatively low sample size, this study analyzed bivariate relations (Pearson Product Correlations) between stalking behaviors, perceived motivations, and personality attributions. We used independent samples t-tests and Chi-Square tests to analyze differences across variables between those victims who were stalked by an (ex-)intimate partner and those who were stalked by others.

## Results

Most participants had (had) an intimate or romantic relationship with the perpetrator (63%). Further, 12% were stalked by a friend, and 4% were stalked by a family member. The remaining participants had experienced stalking from an acquaintance (8%), a colleague (9%), or a neighbor (4%) The duration of stalking perpetration ranged from a minimum of 1 month to a maximum of 28 years. Most participants (17%) had experienced stalking for 2 years and a further 7% had been stalked for over 10 years, and 2% had been stalked for longer than 20 years. The mean duration of stalking perpetration among the participants was 1280.85 days, (3.5 years), with a standard deviation of 1926.28 days (5.3 years). There was no significant difference in duration between those who were stalked in the context of an (ex-) romantic relationship and those in any other context.

The frequencies of stalking behaviors were analyzed, revealing the main stalking behaviors were communication tactics, followed by proximity, violence, other stalking behaviors and lastly, monitoring (See Table 3). Overall, participants experienced a mean of 8.52 stalking behaviors at the hands of the perpetrator (*SD* = 3.21). Participants who were stalked by an (ex-) romantic partner experienced more types of stalking behaviors (*t* (99) = −2.42, *p* = .02) and perpetrators in this context were more likely to use communication methods (*t* (99) = −6.76, *p* < .001) than in all other contexts.

**Table 3.** Frequencies of Stalking Behaviors Experienced by the Victims Split by Relationship.

Stalking behaviors	Total sample%	Ex-intimate	Other	χ^2^
Communication	97%	100%	91%	5.23*
Text messages	83%	94%	65%	13.69***
Contacting friends or family	72%	80%	57%	6.77**
Phone calls	71%	85%	49%	14.25***
Social media contact	64%	72%	49%	6.00*
Emails	53%	72%	22%	23.21***
Violence	76%	83%	65%	3.99*
Threats	50%	48%	51%	.04
Suicidal behaviors or threats	39%	52%	16%	12.82***
Property damage	27%	28%	24%	.21
Physical assault	17%	17%	16%	.03
Sexual assault	11%	11%	11%	<.01
Use of explicit materials	10%	14%	5%	1.38
Proximity	82%	77%	91%	3.89*
Showing up at victim’s home	57%	59%	54%	.21
Following	56%	50%	65%	1.87
Loitering	52%	42%	68%	5.70*
Showing up at the victim’s workplace	43%	39%	51%	1.67
Monitoring	23%	18%	30%	1.50
Digital surveillance	19%	16%	24%	1.08
Tracking device	10%	9%	11%	.04
Other	77%	81%	70%	1.50
Making false allegations	44%	48%	35%	1.87
Unwanted gifts	43%	48%	35%	1.48
Other	31%	25%	41%	2.50

* *p* < .05. **p < .01. ***p < .001.

We then assessed the two motivations for stalking: desire for intimacy and desire to frighten. The correlation between both motivations was negative but significant (*r* = −.24, *p* = .01), suggesting these motivations are not orthogonal. Further, no differences were found between victim-perpetrator relationship for desire to frighten, but those stalked in the context of an (ex-) romantic relationship were significantly more likely to report the perpetrator desired intimacy (*M* = 16.56, *SD* = 3.09) than those in other contexts (*M* = 13.78, *SD* = 4.92; *t* (52.71) = −3.10, *p* = .003).

Following this, we assessed the descriptive statistics of the personality attributions and conducted an independent samples t-tests to assess differences between victims of (ex-) intimate and other stalking. Results are displayed in Table 4. Victims who were stalked by other (non-ex-intimate) people were more likely to report personality attributions of their stalker that aligned with paranoid borderline and dependent PDs.

**Table 4.** Results of Independent Samples *T*-Tests of the Type of Perpetrator-Victim Relationship on the Reported Personality Attributions.

Personality attributions	Sample item	(ex-)intimate		Other		*t*
		*M*	*SD*	*M*	*SD*	
Schizotypal	Does this person have odd beliefs or fantasies that influence behavior?	3.26	.71	3.25	.66	−.10
Schizoid	Does this person lack a desire for intimacy with friends or partners?	3.47	.84	3.33	.80	−.78
Paranoid	Does this person worry excessively about whether they can trust the people close to them?	2.63	.95	3.06	1.03	2.10*
Antisocial	Does this person show recklessness or disregard for the safety of others?	3.02	.79	3.03	.80	.10
Borderline	Does this person display suicidal behavior, threats, or self-mutilating behavior?	2.97	.78	3.50	.55	4.09***
Histrionic	Does this person have an exaggerated expression of emotion?	2.97	.90	2.98	.78	.03
Narcissistic	Does this person need to be admired by others?	2.50	.95	2.62	.94	.64
Dependent	Does this person go to extreme lengths to get support from others?	3.70	.82	4.05	.82	2.10*
Avoidant	Is this person worried about being criticized or rejected?	3.69	.89	3.85	1.01	.83
Obsessive-Compulsive	Does this person pay very close attention to details, rules, lists, order, or organization?	3.65	.85	3.44	.98	−1.14

* *p* < .05. ***p < .001.

Next, we conducted Pearson Product Correlations to examine the association between personality attributions and perceived stalking motivation. Results are displayed in Table 5. Most personality attributions had a significant, positive correlation with a desire to frighten. Only those attributions aligned with avoidant PD showed a positive correlation with a desire for intimacy.

**Table 5.** Pearson Product Correlations Between Motivation to Stalk and Personality Attributions.

Personality attributions	Desire for intimacy	Desire to frighten
Schizotypal	.06	.43***
Paranoid	.13	.42***
Schizoid	−.03	.37***
Antisocial	−.29**	.66***
Borderline	.19	.43***
Histrionic	−.06	.56***
Narcissistic	−.17	.67***
Dependent	.19	.17
Avoidant	.32*	.08
Obsessive-compulsive	.06	.37***

* *p* *<* .05.** *p* *<* .01. ***p < .001.

We then correlated different types of stalking behaviors with personality attributions as reported by the victim. Results are displayed in Table 6. Overall, stalkers with higher Cluster B-aligned personality attributions were more likely to engage in violent behaviors, and to engage in more stalking behaviors. Interestingly, stalkers with Schizotypal or Paranoid-aligned personality attributions were also more likely to engage in violent behaviors. None of the personality attributions were significantly related to monitoring behaviors.

**Table 6.** Pearson Correlations Between Reported Personality Attributions and Types of Stalking Behaviors.

Personality Attributions	Communication	Violence	Proximity	Monitoring	Other	Total
Antisocial	.07	.41***	.20*	.12	.06	.33**
Borderline	.33**	.30**	.06	−.06	.08	.30**
Histrionic	.11	.29**	.23*	.02	.08	.30**
Narcissistic	.22*	.29**	.31**	.19	.30**	.46***
Schizotypal	.20*	.21*	.06	−.08	.14	.22*
Schizoid	.19	.10	.07	.02	.19	.20*
Paranoid	.33**	.22*	<.01	.04	.01	.24*
Dependent	.22*	.01	−.05	−.04	.06	.08
Avoidant	.21*	.07	−.07	−.03	−.03	.07
Obsessive-compulsive	.23*	.11	.09	.10	.21*	.25*

* *p* < .05. **p < .01. ***p < .001.

Finally, we conducted Pearson correlations to examine the association between motive to stalk and types of stalking behaviors engaged in. Results are displayed in Table 7.

**Table 7.** Pearson Correlations Between Stalking Motivation and Types of Stalking Behaviors.

Desire	Communication	Violence	Proximity	Monitoring	Other	Total
Intimacy	.19	−.05	−.06	−.07	−.15	−.01
Frighten	.23*	.41***	.27**	.22*	.21*	.48***

* *p* < .05. **p < .01. ***p < .001.

## Discussion

This study found a significant relationship between personality attributions, stalking behaviors, and motivation to stalk as reported by victims. As hypothesized, personality attributions aligned with Cluster B PDs were most common, supporting Mullen et al.’ (1999) claims. Specifically, perpetrators were perceived to have the most personality attributions aligned with Narcissistic PD, followed by Antisocial PD. The results support and confirm Sansone and Sansone’s (2010) finding that the prevalence of PD features is substantially higher where being charged with a stalking offence is not necessary. Although personality attributions aligned with Cluster B PDs were most common among the stalkers, the present study has highlighted some interesting findings that are often neglected in the study of stalkers and psychopathology. Consistent with Nijdam-Jones et al. (2018), the present study aimed to expand the focus and found that personality attributions related to Paranoid, Obsessive-Compulsive and Schizotypal PDs were most commonly reported Cluster A and C PD attributions. Further, those who had been or still were in a romantic relationship with the perpetrator were more likely to report attributions aligned with Borderline and Paranoid PD than those stalked by anyone else.

We developed a measure to assess motivation to stalk, and found that there was no difference in motivation to frighten between ex-intimates and all other relationships, but ex-intimates were more likely to report a desire for intimacy on the part of their stalker. Further, and consistent with previous research, a desire to frighten was positively associated with all personality attributions aligned with Cluster B PDs whereas a desire for intimacy was negatively correlated with antisocial PD-aligned attributions. Interestingly, desire to frighten was also significantly correlated with most other personality attributions. Furthermore, there was a significant positive correlation between Avoidant personality attributions and desire for intimacy. Individuals with avoidant traits may have an active desire for relationships, but fear of rejection and criticism due to feelings of social inadequacy, resulting in feelings of loneliness (Lampe & Malhi, 2018). These individuals may lack relationship skills due to their avoidant nature so they may try to initiate relationships in an inappropriate manner, with or without intent to cause fear.

Further, the results highlighted that nearly all the victims experienced communicative and proximity behaviors, supporting previous research into the escalation of stalking as McEwan et al. (2012) found that communication escalated to proximity seeking behaviors in the majority of stalkers. Repetitive communication and proximity seeking behaviors can be intrusive and have long-lasting harmful effects on the victims as victims can feel they have no place to hide and can be constantly fearful of their stalkers turning up at their home or place of work (Korkodeilou, 2017). Participants who were stalked by an (ex-) romantic partner reported more types of stalking behaviors, and particularly, a greater use of communication methods. It is likely that more behaviors are available to a stalker in this context, for example, (prior) access to social media, phone number(s), address, etc. Alarmingly, three quarters of these victims experienced some form of violence at the hands of their stalkers, most notably threats of suicide, thus raising the question whether stalking behaviors such as proximity stalking can lead to violent behaviors if there is no intervention from police. These results highlight the severity of stalking and the need for police and criminal justice professionals to take early intervention on what they may view as “harmless” behavior, such as texting or being spotted outside the victim’s home/workplace as there appears from the results to be a clear pattern of violence among stalkers. Unfortunately, the sample size did not allow for an exploration of the association between time and escalating use of stalking behaviors. Future research may wish to explore such a link.

Finally, we expected to see a distinction in stalking behaviors perpetrated based on motivation to stalk. The results revealed a clear distinction between behaviors perpetrated by stalkers with a desire to frighten, compared with stalkers who desire intimacy with their victims. The latter was more likely reported by someone who was stalked by an (ex-) romantic partner. Stalkers who were motivated by a desire to frighten were significantly more likely to carry out all the stalking behaviors examined, and they were more likely to perpetrate the greatest number of stalking behaviors. Out of all the stalking behaviors, motivation to frighten was most significantly correlated with violence and proximity behaviors—suggesting that victims fear in this regard should be taken very seriously by police when incidents of stalking are reported.

## Implications

The present study has potentially important implications. This study offers a new insight into stalking perpetration, motivation, and psychopathology of stalkers for those who work with victims. Previous research has suggested a need to develop up-to-date and mandatory training for criminal justice workers and those who support victims of stalking (Ngo, 2020; Weller et al., 2013). From the study’s findings, stalkers at a potentially increased risk of committing violence are those are perceived to possess personality traits aligned with Antisocial PD. Along with this, all the other personality attributions aligned with category B PDs were strongly correlated with violent behavior, such as physical assault or threats, as well as a correlation between personality attributions related to Paranoid PD, Schizotypal PD, and violent behaviors. These findings can have important applications for those who work with stalkers or their victims, suggesting it is essential for these individuals to be aware of personality features that are associated with, or predictors of violence among stalkers. Furthermore, personality attributions aligned with Narcissistic PD significantly predicted proximity stalking behaviors, meaning those who work with victims on stalking should be wary for narcissistic traits and warn the victims to ensure personal safety and security to minimize this type of stalking, particularly as proximity stalking behaviors such as following have been found to elicit the most fear in victims compared to other stalking methods (Dietz & Martin, 2007).

The present study also examined personality attributions and their association with stalking behaviors. This is an area for future research to develop as the study found for the first time a significant relationship between stalking behaviors and personality attributions that are potentially more likely to be dismissed by the police, or perhaps are more likely to go undetected. The significant relationship between desire to frighten and personality attributions aligned with Obsessive-Compulsive PD is of importance. It was predicted in the present study that some of the stalkers would have personality attributions aligned with Obsessive-Compulsive PD as these individuals often become angry and frustrated when their need for control and perfectionism is not realized (Darjee, & Davidson, 2010), potentially triggering a motivation for stalking, as a means for establishing control (Johnson, & Hotton, 2003). These individuals may go undetected in studies using forensic samples, as these individuals are meticulous and calculated and may go under the radar more than individuals with Cluster B traits that involve impulsivity and emotional dysregulation, avoiding detention. Moreover, Obsessive-Compulsive PD-related personality attributions were related to violent behaviors. This finding highlights the need for professionals such as the police and victim support to receive training to aid in the understanding of the relationship between personality traits and stalking, in order to recognize features that are associated with violent stalking that often go under the radar.

The present study also found a wide variety of stalking behaviors reported by the victims. The results have shown that almost all the victims experienced communicative stalking, such as multiple texts or phone calls and almost all the victims had also experienced proximity stalking behaviors. This highlights the worrying nature of victims being told to change their own behavior to try and minimize the behaviors experienced at the hands of the stalker (Spitzberg, 2002; Storey & Hart, 2011), such as changing their numbers or their route to work as their stalkers may be motivated by their obsession to find a new way to stalk, which could lead to an escalation in behaviors. As three quarters of the victims had experienced violence, this clearly highlights the importance of early intervention, before an escalation to violence as previous research has demonstrated the widespread effect on victims’ lives (Taylor-Dunn et al., 2021). It would perhaps be of benefit for future research to explore the temporal orders of the behaviors explored within this study, as it would be beneficial to understand whether there is a clear escalation of behaviors among stalkers, depending on their motivations to stalk. Future research should investigate behaviors and common personality traits among stalkers in the early stages of stalking perpetration, or in retrospect before the stalking began, to provide an insight into potential early warning signs of stalking.

The study poses the question of whether the stalker’s motivation to cause fear in the victim should be taken more seriously among police and criminal justice workers. Previous research has highlighted victims often report inaction, or inappropriate action taken by the police, with stalking being dismissed as harassment—in addition to feeling blamed and not being taken seriously (Taylor-Dunn et al., 2021). Victims have described feeling as though they are not taken as seriously if they do not appear frightened of imminent violence or have not yet experienced violence (Korkodeilou, 2014). However, if the perpetrator is intending to frighten their victim, the present study suggests that this alone should be taken seriously as stalkers motivated to frighten their victims are significantly more likely to perpetrate the most stalking behaviors, including violent behaviors, which causes complex and often traumatic, long-term psycho-social effects on victims (Korkodeilou, 2017). The present research suggests that criminal justice workers should be trained in personality traits common among stalkers that can be associated with motivations to cause fear, to better protect victims of stalking.

## Limitations

A limitation of the present study is the use of self-report data from the victim’s perspective. Although this offers a unique perspective, the victims of stalking may not be the best judges of their stalker’s behavior. For example, motivations for stalking were assessed based on the victim’s opinions, such as “Does this person stalk with the hope of intimacy?” or “Does this person enjoy the sense of control that comes from stalking?” The victims may not know the answer to this question. Furthermore, it is plausible that the victims do not know the full extent of the stalker’s behavior, such as more discrete monitoring behaviors—potentially explaining why the monitoring behaviors were the least common among the stalkers in the present study. Thus, this raises an issue as to how well the victims can judge their stalkers’ motivation and behavior. Nevertheless, the victim’s experience of the stalker and their behavior provides a valuable perspective.

A further limitation is that the sample only included victims who reported being stalked and sought help via the National Stalking Helpline. It is plausible that those who reported being stalked experienced a course of conduct that made them more fearful than those who have been stalked and did not report it or they may have felt that their stalking victimization may be taken more seriously. Indeed, Reyns and Englebrecht (2014) found that seriousness and victims’ fear increased the probability of reporting stalking, perhaps due to victims being dismissed by police if they are not deemed “fearful enough” (Reyns & Englebrecht, 2013). Previous research has found that victims tend to be more fearful of stalking by strangers, as opposed to acquaintances and intimate partners (Podaná & Imríšková, 2016), despite indications that stalking by an (ex-) intimate partner is most likely to result in extreme harm or death (McFarlane et al., 2002). Furthermore, a stalking victim may view the behavior of someone known to them as less blameworthy, and therefore less serious, making reporting stalking unwarranted. Yet, because we know that individuals are more likely to be stalked by someone known to them (Douglas & Dutton, 2001; Jordan et al., 2007), underreporting to the police becomes a significant issue. As a result, it is likely that the perspectives of victims who view their stalking as less “serious” or are less fearful are not included within the study, and thus results may not be generalizable to all victims of stalking. In a similar vein, the victims in this study were self-identified victims, and we did not use legal or empirical recommendations as additional inclusion criteria.

A final limitation is that the results from the present study are not generalizable to all stalkers. The study specifically focused on stalkers who were known to the victim, such as or (ex-)partners, friends, family, or acquaintances. As such, the present study was unable to obtain results on stalkers who do not know their victims. Previous research has highlighted that compared with intimate and acquaintance stalking, stranger stalking, or the stalking of public figures is likely to result from severe mental illness, such as psychotic illness (Mohandie et al., 2006).

## Conclusion

This was an exploratory study that used a convenience sample of self-identified stalking victims. The results of the present study indicate the need for the police and criminal justice professionals to receive up to date training on stalking, exploring the association between stalking motivations, behaviors perpetrated, and personality disorder features. It underlines the magnitude of stalking, particularly among those who are stalked in the context of an (ex-) romantic relationship. The present study highlights the importance of expanding the focus beyond the most well-known and prevalent personality traits among stalkers. Further, the results highlight the severity of stalking behaviors perpetrated by stalkers with personality attributions aligned with Cluster B PDs and paranoid, schizotypal, and obsessive-compulsive PDs. The present study demonstrates the need for professionals such as police and victim support to understand the relationship between stalking, personality attributions, and motivation to stalk in to better assist victims in recognizing such features displayed by their stalkers, as well as to protect victims from the risks associated with this.
